# A multinodular goiter as the initial presentation of a renal cell carcinoma harbouring a novel *VHL *mutation

**DOI:** 10.1186/1472-6823-6-6

**Published:** 2006-10-26

**Authors:** Maria João M Bugalho, Evelina Mendonça, Patrícia Costa, Jorge Rosa Santos, Eduardo Silva, Ana Luísa Catarino, Luís G Sobrinho

**Affiliations:** 1 Serviço de Endocrinologia, Instituto Português de Oncologia Francisco Gentil, Lisboa, Portugal; 2 Centro de Investigação de Patobiologia Molecular, Instituto Português de Oncologia Francisco Gentil, Lisboa, Portugal; 3 Serviço de Anatomia Patológica, Instituto Português de Oncologia Francisco Gentil, Lisboa, Portugal; 4 Serviço de Cirurgia de Cabeça e Pescoço, Instituto Português de Oncologia Francisco Gentil, Lisboa, Portugal; 5 Serviço de Urologia, Instituto Português de Oncologia Francisco Gentil, Lisboa, Portugal

## Abstract

**Background:**

Secondary involvement of the thyroid gland is rare. Often the origin of the tumor is difficult to identify from the material obtained by fine-needle aspiration cytology. Renal cell carcinoma of the clear-cell type is one of the more common carcinomas to metastasize to the thyroid gland. Somatic mutations of the von Hippel-Lindau tumor suppressor gene are associated with the sporadic form of this tumor. We aimed to illustrate the potential utility of DNA based technologies to search for specific molecular markers in order to establish the anatomic site of origin.

**Case Presentation:**

A 54-yr-old Caucasian male complaining of a rapidly increasing neck tumor was diagnosed as having a clear-cell tumor by fine-needle aspiration cytology. A positive staining for cytokeratin as well as for vimentin and CD10 in the absence of staining for thyroglobulin, calcitonin and TTF1 suggested a renal origin confirmed by computed tomography.

Using frozen RNA, obtained from cells left inside the needle used for fine needle aspiration cytology, it was possible to identify a somatic mutation (680 delA) in the *VHL *gene.

**Conclusion:**

In the presence of a clear-cell tumor of the thyroid gland, screening for somatic mutations in the *VHL *gene in material derived from thyroid aspirates might provide additional information to immunocytochemical studies and therefore plays a contributory role to establish the final diagnosis. Moreover, in a near future, this piece of information might be useful to define a targeted therapy.

## Background

Metastasis to the thyroid is an uncommon occurrence. In clinical series, the frequency of secondary involvement of the thyroid is considerably lower than the frequency of 25% found at autopsy of patients with disseminated malignancies [1,2].

The increasing number of reported cases of secondary tumors to the thyroid, as suggested by two studies from the Mayo Clinic referring to distinct periods [1,3], is probably related with routine evaluation of thyroid nodules in patients with known cancer by fine-needle aspiration cytology (FNAC) [4]. Nonetheless, the diagnosis of secondary involvement of the thyroid gland in a patient with an asymptomatic primitive neoplasm is still challenging.

Secondary involvement of the thyroid gland by renal cell carcinoma (RCC) clear-cell type (CCRCC) is rare; however CCRCC is one of the more common neoplasms to metastasize to the thyroid gland [5]. Since metastatic clear-cell RCC must be distinguished from a variety of neoplasms with clear-cell features including primitive tumors of thyroid, interpretation of FNAC may be a diagnostic problem for the pathologist [6,7] and imposes the combination of routine immunohistochemical studies. A different approach might be the use of DNA based technologies. Screening for molecular markers or somatic mutations known to be specific of particular tumors appears as a promising tool to refine diagnosis. In this setting, considering that the von Hippel-Lindau tumor suppressor gene (VHL) is mutated or silenced in more than 50% of sporadic renal cell carcinomas of the clear cell type [8-12], identification of a mutation in the *VHL *gene in DNA derived from the leftover cells in the needle used for FNAC might be helpful to establish the origin of the primitive tumor.

## Case presentation

### Case history

A 54-yr-old Caucasian male was referred for evaluation of a rapidly increasing neck tumor noticed for the first time four months earlier. He complained of sporadic dysphagia without weight loss. He was a heavy smoker until recently but had no previous medical history. He worked as a house painter, for seven years, in his thirties. The physical examination revealed an asymmetric multinodular goiter with a left dominant nodule with firm consistency and no palpable regional nodes. Serum TSH was 1.3 μUI/ml (normal: 0.5–4.7 μUI/ml), T4 7.3 μg/dl (normal: 5.4–11 μg/dl) and T3 126 ng/dl (normal: 52–160 ng/dl). Antimicrosomal and antithyroglobulin antibodies were negative. A thyroid ultrasound demonstrated a multinodular gland with the right and left lobes measuring 6 × 4 × 3 cm and 9 × 7 × 6 cm respectively. A computed tomography (CT) scan of the neck showed a slight tracheal deviation to the right without compression. A Tc-99 m scintigraphy disclosed irregular uptake in both lobes of the thyroid and a large cold nodule in the left lobe. FNAC from both lobes revealed a clear-cell carcinoma with an immunocytochemical profile suggestive of a secondary tumor from the kidney (refer to section pathology).

FNAC results prompted a clinical and radiographic investigation. An abdominal CT scan revealed a tumor of the left kidney measuring in greatest diameter 10 cm. Bone scan, chest computed tomography scan, liver ultrasound and laboratory data were normal and there was no evidence of other distant metastases.

Initial treatment included a left radical nephrectomy and a total thyroidectomy. One year later, there was evidence for cervical nodal metastases. The patient was then submitted to right radical neck dissection with internal jugular vein ligation and section of spinal accessory nerve and left modified radical neck dissection type III. At histological examination, only right nodes were metastatic.

### Pathology

Both thyroid lobes were sampled. Smears were air dried and acetone fixed and stained with May-Grünwald-Giemsa (MGG) and Papanicolaou (PAP) stains, respectively. An additional sample was fixed in formalin and processed as a cell-block using the Shandon Cytoblock^® ^Kit (Thermo Electron Corporation, Pittsburgh, PA, USA). Cell block (CB) sections were stained with hematoxilin-eosin. Immunocytochemistry was performed on CB sections, using an avidin-biotin method with diaminobenzidine as the chromogen for the following antibodies: thyroglobulin, calcitonin, vimentin, CD10, TTF1 (Dakocytomation, Denmark A/S) and cytokeratin AE1/AE3 (Zymed Laboratories, Inc, San Francisco, CA).

**Figure 1:**
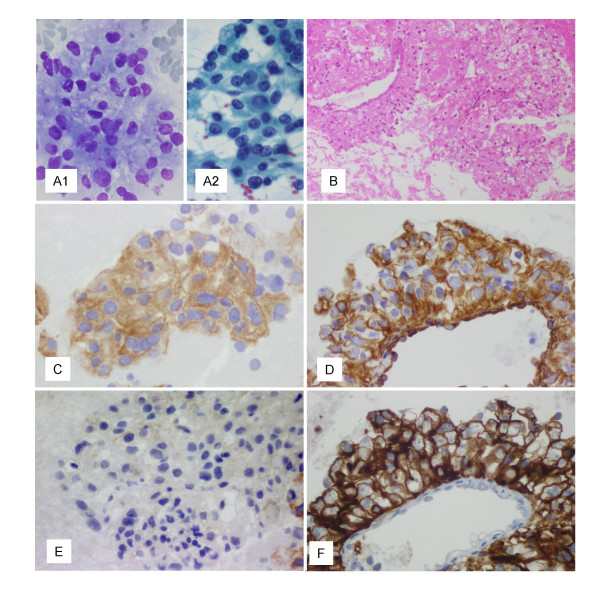
A – Tumor cells with clear cytoplasm, large atypical nuclei, conspicuous nucleoli (A1 – MGG; ×400; A2 – PAP; ×400). B – Cellblock section. Prominent vascular pattern (H&E; ×100). C – Cell block. Immunoreactivity for AE1/AE3 (×400). D – Cell block. Immunoreactivity for vimentin (×400). E – Cell block. Negative immunostain for TTF1 (×400). F – Cell block. Strong surface membrane staining for CD10 (×400).

Smears consisted of a clear, large cell neoplasia with large nuclei, prominent nucleoli and finely vacuolated cytoplasm, with indistinct borders. The tumor cells were arranged in aggregates of variable size and shape, many of them centered by thin walled capillaries. This intimate relationship of neoplastic cells and vessels was better appreciated on cell-block sections. These cells were immunoreactive for pancytokeratin (AE1/AE3), vimentin and CD10 and were negative for thyroglobulin, thyroid transcription factor1 (TTF1) and calcitonin (Fig. 1).

The smear pattern together with the immunocytochemical profile was consistent with the diagnosis of secondary tumor, most probably from renal origin.

The nephrectomy specimen showed a renal cell carcinoma, clear cell type. It was classified as Fuhrman 3 and showed extra renal local spread and no vascular invasion.

The thyroid gland was multinodular and all the nodules consisted of metastasis of a clear cell neoplasia. The histological pattern was similar to the renal tumor. Immunocytochemical study was performed using the same antibodies that were tested on cytological samples with identical results.

### *VHL *mutation analysis

RNA from cells left inside of the needle used for FNAC was isolated with the QuickPrep micro mRNA Purification Kit (Amersham Pharmacia Biotech, Buckinghamshire, UK), according to the manufacturer's instructions. Half of the RNA was reversed transcribed with Superscript (Invitrogen Corporation, Carlsbad, CA, USA) in 20 μl reaction volume with random primers and cDNA kept frozen. To screen for VHL mutations in thyroid aspirates, 2,5 μl of first-strand cDNA was used as a template for PCR using primers designed by us (F-5'-TCAGAGATGCAGGGACACAC-3', R-5'-TGACGATGTCCAGTCTCCTG-3').

Somatic DNA was extracted from samples corresponding to renal carcinoma and thyroid metastases obtained during surgeries and immediately frozen with liquid nitrogen until nucleic acid extraction using TRIzol Reagent (Life Technologies, Inc., Gaithersburg, MD, USA). Genomic DNA was also obtained from peripheral venous blood and isolated by a manual method adapted from Bowtell [13].

DNA samples were amplified by PCR using primers previously described [14]. The screening of *VHL *mutations was performed by single-strand conformational polymorphism analysis (SSCP). To further characterize the abnormal pattern observed in the SSCP, PCR purified products were either sequenced directly using the ABI PRISM^® ^BigDye™ Terminator Cycle Sequencing Ready Reaction Kit (Applied Biosystems, Foster City, CA, USA) and the ABI PRISM 310 Genetic Analyser or subcloned into pGEM^®^-T Easy Vector (Promega, Madison, USA), and subsequently sequenced using the ABI PRISM^® ^BigDye™ Terminator Cycle Sequencing Ready Reaction Kit (Applied Biosystems, Foster City, CA, USA) and the ABI PRISM 310 Genetic Analyser. Restriction analysis, using the restriction endonuclease BstZ17 I (New England BioLabs^®^, Inc., Beverly, USA) was also performed.

**Figure 2:**
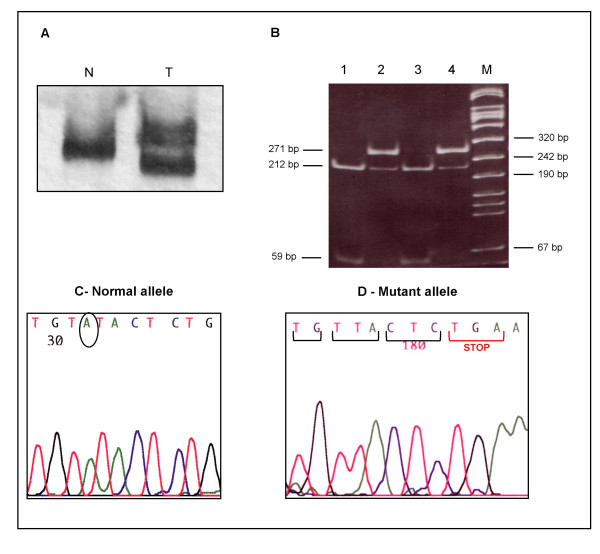
A – Non-radioactive PCR-SSCP analysis of *VHL *exon 3; T – altered mobility observed in a tumor sample, relative to the normal control N. B – Ethidium bromide-stained polyacrylamide gel of BstZ17 I restriction digestion. Lane 1 – peripheral venous blood, Lane 2 – renal cell carcinoma, Lane 3 – non tumoral tissue from kidney, Lane 4 – thyroid metastases, M – denotes the lane containing the pUC Mix Marker 8 (Fermentas, Burlingyon, Canada). For the wild allele, restriction digestion produces bands of 212 and 59 bp. The mutation abolishes the restriction site and the mutant allele corresponds to a 271 bp band. C and D – Tumour DNA sequence in the *VHL *codon 156 region of cloned exon 3 amplicons. As a consequence of the 680 delA, a premature stop codon appears in the mutant allele.

The same alteration, a heterozygous 680delA (codon 156/exon 3) of the *VHL *gene was identified in thyroid aspirates, renal carcinoma, thyroid metastases and lymph node metastases. It causes a frame-shift and creates a premature stop predicting a truncated pVHL (Fig. 2). Constitutional DNA (peripheral venous blood) was analyzed and did not show the mutation. No other alterations were observed in exons 1 and 2 of *VHL*.

## Conclusion

Metastatic disease to the thyroid gland from distant sites is rare. Breast and lung carcinoma are the most frequently identified sources of secondary thyroid carcinoma found at autopsy while renal cell carcinoma comprises over 50% of secondary thyroid malignancies discovered clinically [15]. Clear-cell RCC is the most common malignant neoplasm of the kidney [16]. Although the kidney is the most common primary anatomic site for clear-cell tumor, other primary sites include the lung, salivary gland, breast, ovary, endometrium, cervix, vagina, pancreas, liver, adrenal gland and thyroid [6].

Metastases of a clear-cell tumor present similar morphologic features regardless the origin of the primitive tumor. In a patient with a past history of malignancy elsewhere, the finding of a clear-cell tumor on thyroid aspirates will prompt the hypothesis of a secondary tumor. The sudden enlargement of the thyroid in an otherwise healthy individual, as it was the case in our patient, makes the diagnosis of metastatic disease challenging for the cytopathologist and reinforces the need of immunohistochemical studies [6,7]. A positive staining for cytokeratin as well as for vimentin and CD10 in the absence of staining for thyroglobulin, calcitonin and TTF1, observed in smears from both lobes of the thyroid, favored the hypothesis of metastasis from a renal carcinoma. These findings guided the subsequent diagnostic workup and a hitherto occult neoplasm of the left kidney was documented. Curiously, from literature, the left kidney seems to be more affected [5].

RCC is a highly treatment-resistant tumor type and surgery remains the standard approach. Pending the extent of the metastatic disease and the general condition of the patient, the surgical management of metastases is justified as long term survivals have been reported [17-19]. Considering the solitary nature of thyroid metastases in our patient, after a radical nephrectomy a total thyroidectomy was performed.

Since somatic *VHL *mutations account for the majority of sporadic CCRCCs [8,12] and may represent a prognostic factor [12] we sought for mutations of the *VHL *gene in different samples of tumor DNA including frozen cDNA obtained from the cells left inside the needle used for fine-needle aspiration cytology. cDNA had been obtained and kept frozen in the setting of an independent ongoing study to seek for molecular markers in thyroid aspirates.

The same heterozygous mutation, a 680 delA, (codon 156/exon 3), was observed in all tumor samples and absent in constitutional DNA. The tumor was therefore defined as sporadic. Although a broad spectrum of somatic *VHL *mutations has been described in patients with CCRCCs, mutations cluster within exon 2 [8]. To the best of our knowledge, the mutation we identified in the present case was not previously reported [8,9,11,12,20,21]. Non-random distribution of somatic *VHL *mutations may originate from exposure to exogenous carcinogens [22]. According to the 2-hit theory of tumor suppressor genes, another hit in *VHL *might be involved. Hypermethylation studies were not conducted thus we can not exclude inactivation of the wild type allele and on the other hand, contamination with normal tissue can not be ruled out. Despite the previous comment, the frequency of biallelic inactivation of *VHL *in sporadic renal cell carcinomas was found to be unexpectedly low [23].

The possibility to screen for *VHL *mutations in thyroid aspirates may help in distinguishing metastatic renal cell carcinoma from other primary clear-cell tumors. *VHL *mutations have not been identified in the thyroid except in MEN 2 associated medullary thyroid carcinoma [24] although LOH in the *VHL *gene has been identified in follicular carcinomas of the thyroid [25]. Herein, we used frozen cDNA obtained from cells left inside the needle used for FNAC. Contamination by normal thyroid cells, as verified by thyroglobulin expression (data not shown), did not blur the results.

The *VHL *gene regulates the oxygen-dependent expression of genes involved in the cellular response to oxygen deprivation. *VHL *interacting with the transcription factors hypoxia inducible factors HIF 1α and HIF 2α mediates transcriptional activation of genes involved in angiogenesis, erythropoesis and anaerobic metabolism. Given that multiple pathways contribute to tumor growth, it is conceivable that anti-tumor activity may be increased by agents targeting multiple pathways [26-28].

Thus, identification of a somatic mutation in the *VHL *gene may be helpful to establish a diagnosis as well as to make a decision concerning emerging targeted therapies [29].

## Abbreviations

CCRCC – clear cell renal cell carcinoma; FNAC – fine needle aspiration cytology; LOH – loss of heterozygosity; MEN 2 – multiple endocrine neoplasia type 2; RCC – renal cell carcinoma; VHL – von Hippel Lindau

## Competing interests

The author(s) declare that they have no competing interests.

## Authors' contributions

MJB – Provided clinical care for the patient, coordinated studies and draft the manuscript; EM – cytopathologist who carried out the immunocytochemical studies and interpreted results; PC – carried out the molecular genetic studies; JRS – conducted total thyroidectomy; ES – conducted the nephrectomy; ALC – performed the histopathological analysis; LS – critical revision.

All the authors read and approved the final manuscript.

## Pre-publication history

The pre-publication history for this paper can be accessed here:


